# Supplementary material for: Global kinome profiling reveals DYRK1A as critical activator of the human mitochondrial import machinery
Source: Nat Commun. 2021 Jul 13;12:4284. doi: 10.1038/s41467-021-24426-9 (PMC8277783; doi:10.1038/s41467-021-24426-9)
Supplement: Supplementary file 1 — Supplementary Information [file 41467_2021_24426_MOESM1_ESM.pdf]

Supplementary Information

Supplementary **Figures 1-6**

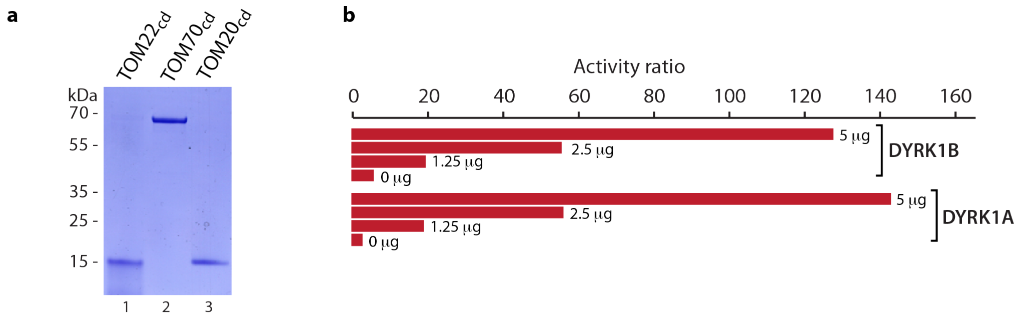

c

Protein	Peptide	Peptidesite	Probability	Proteinsite
TOM70	KTPEGRASPAPGSGHPEGPGAHLDMNSLDR	2	T(2): 95.5; S(8): 4.2; S(13): 0.2; S(27): 0.0	T85
TOM70	ASPAPGSGHPEGPGAHLDMNSLDR	2	S(2): 100.0; S(7): 0.0; S(21): 0.0	S91
TOM70	ASPAPGSGHPEGPGAHLDMNSLDR	2, 21	S(2): 97.8; S(7): 2.2; S(21): 100.0	S91, S110

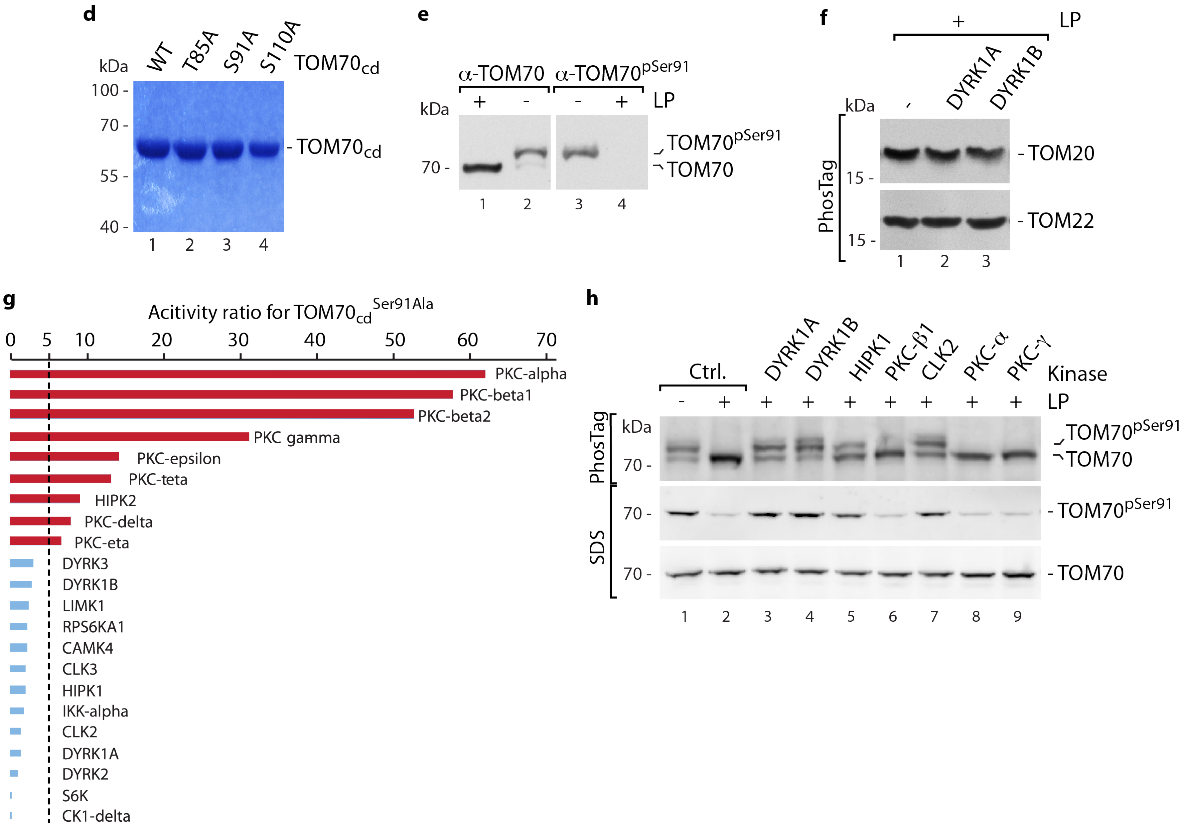

Supplementary Figure 1 | In vitro and in vivo phosphorylation of TOM70^{Ser91} by DYRK1A and DYRK1B. a, Coomassie staining of SDS-PAGE loaded with soluble receptor domains of human TOM22$_{cd}$, TOM70$_{cd}$ and TOM20$_{cd}$. **b**, Activity ratios of ^{33}P-ATP-radiometric filter binding assays for DYRK1A and DYRK1B in the presence of increasing concentrations of TOM70$_{cd}$ protein. **c**, Overview of the three TOM70 phosphorylation sites that were identified in HEK293T cells (see Methods). **d**, Coomassie staining of SDS-PAGE loaded with indicated human TOM70$_{cd}$ variants with mutations to non-phosphorylatable alanine residues. **e**, Phos-tag gel analysis of TOM70 in isolated mitochondria incubated in

the absence (-) or presence (+) of LP and analysis with TOM70 and $TOM70^{pSer91}$ specific antisera. **f**, Phos-tag gel analysis of TOM20 and TOM22 receptors in isolated mitochondria that were incubated with DYRK1A and DYRK1B after LP treatment (as shown for TOM70 in Figure 1d, lanes 2-4). **g**, Results of *KinaseFinder* assay performed for $TOM70_{cd}^{Ser91A}$ variant. Assay and data processing was performed as for $TOM70_{cd}^{WT}$ (Fig. 1a). Dashed line indicates threshold for activity ratios[46]. **h**, Re-phosphorylation of isolated mitochondria that were treated with LP and incubated with indicated kinases. Experiment was performed as in Fig. 1d. Unprocessed immunoblots and gels are reported in the Source Data File.

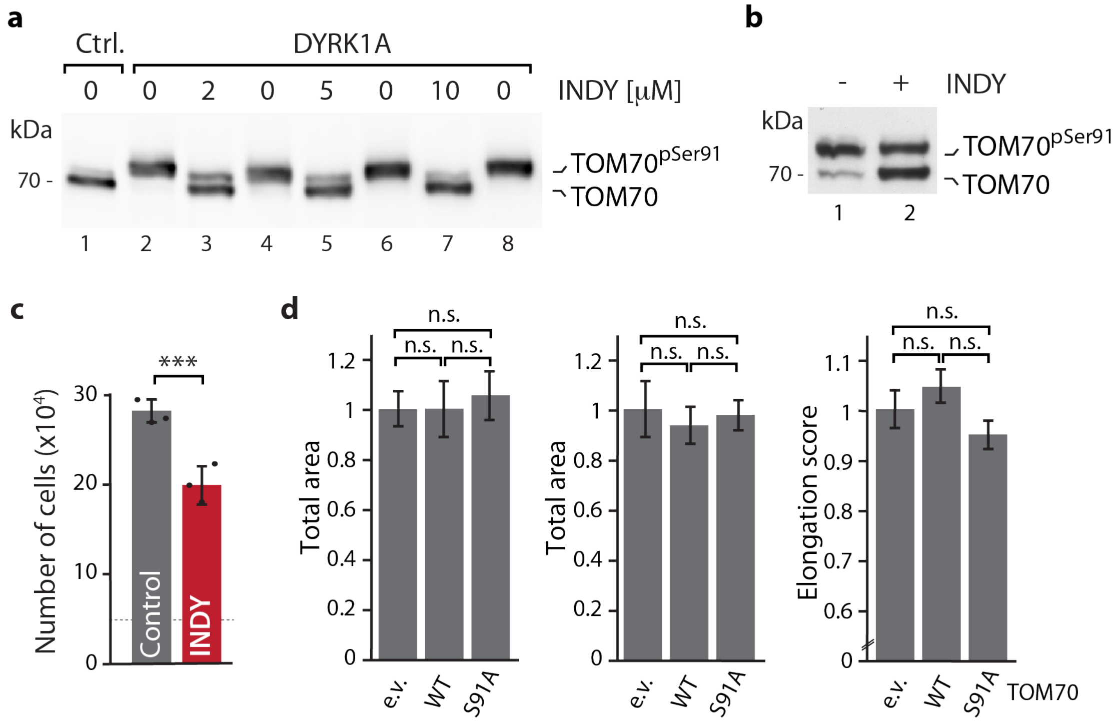

Supplementary Figure 2 | Inhibition of DYRK1A signaling by INDY. a, Titration of INDY to test efficient concentrations for inhibition of $TOM70_{cd}$ phosphorylation by DYRK1A. Samples were analyzed via Phos-tag gel analysis and immunoblotting using TOM70 antisera. **b**, Phos-tag gels of mitochondria isolated from cells grown overnight in the presence or absence of INDY (10 μM). Samples were analyzed as in (**a**). **c**, Impairment of cell growth by treatment with INDY (10 μM) for 48 h. Control, DMSO only. Dashed line reflects starting cell number. Data represent mean ± SEM from three independent experiments. Statistical analysis was performed using a Student's t-test (p=0.0035). **d**, Quantification of microscopy data from overexpression of $TOM70^{WT}$ and $TOM70^{Ser91Ala}$ variants compared to empty vector (e.v.) as control (n=16 cells). Data represent mean ± SEM. Statistical analysis was performed using a one-way ANOVA followed by a Bonferroni post-hoc test to allow multiple comparisons. n.s., not significant (*p* > 0.05). Numerical source data and unprocessed immunoblots are reported in the Source Data File.

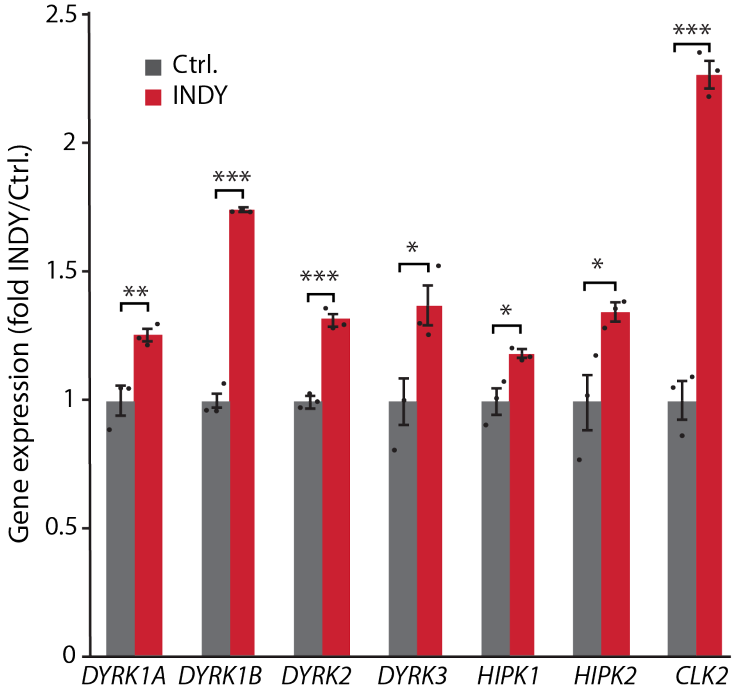

Supplementary Figure 3 | Inhibition of DYRK1A by INDY activates expression of further DYRK family kinases. Analysis of changes of indicated transcript levels upon DYRK1A inhibition (INDY treatment, 10 µM) by qRT-PCR. n = 3 (technical replicates), data represent mean ± SEM and are representative of two independent experiments. Statistical analysis was performed using a two-sided Student's t-test to compare between two groups (p=0.0083 (*DYRK1A*); p=0.00002 (*DYRK1B*); p=0.0002 (*DYRK2*); p=0.0288 (*DYRK3*); p=0.0169 (*HIPK1*); p=0.0291 (*HIPK2*); p=0.0001 (*CLK2*)). Numerical source data are reported in the Source Data File.

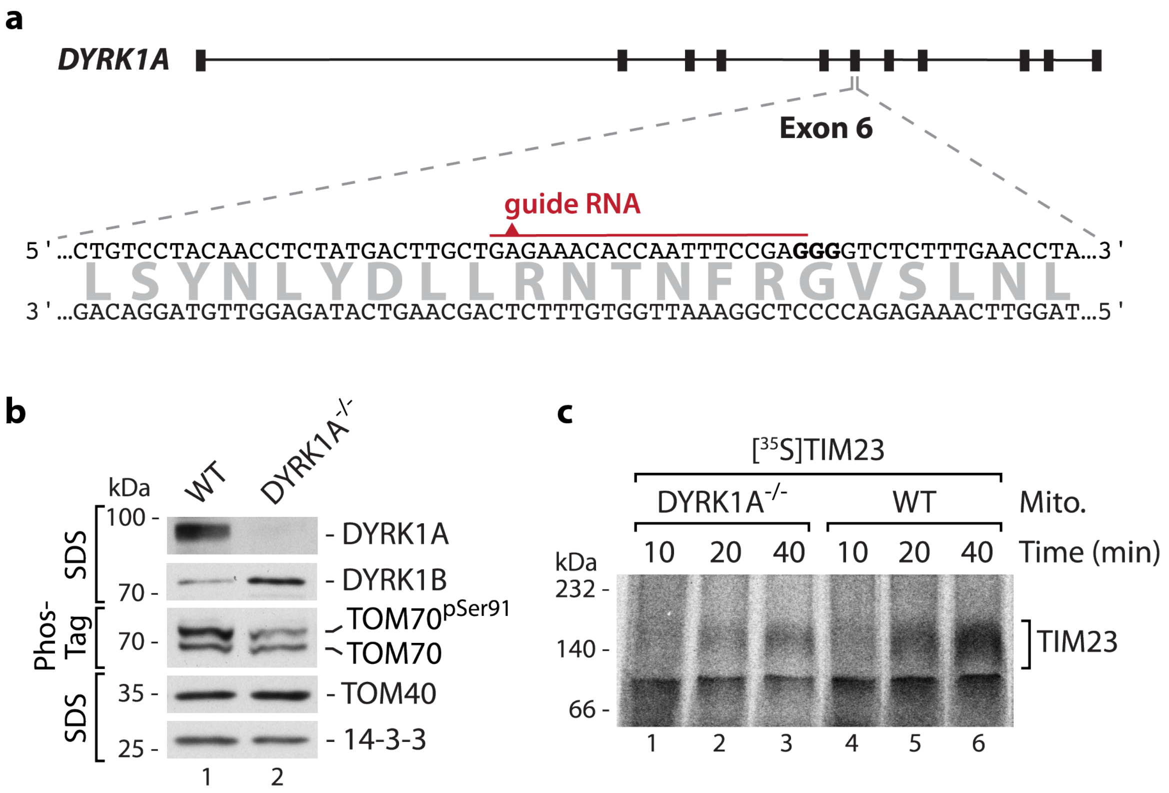

Supplementary Figure 4 | Inhibition of carrier import in mitochondria from *DYRK1A$^{-/-}$* cells. a, Strategy to target human *DYRK1A* by CRISPR-Cas9. Guide RNA was directed against exon 6 of *DYRK1A*. Successful targeting of the *DYRK1A* gene was confirmed by sequencing and immunodecoration. **b**, Western blot analysis of wild-type (WT) and *DYRK1A$^{-/-}$* cells after SDS-PAGE reveals absence of immunoreactive DYRK1A signal and increased level of DYRK1B. Phos-tag analysis revealed decrease of TOM70^{Ser91} phosphorylation. 14-3-3, loading control. **c**, Import of radiolabelled TIM23 precursor into isolated mitochondria from WT and *DYRK1A$^{-/-}$* cells analysed by Blue Native PAGE. Unprocessed immunoblots and autoradiography scans are reported in the Source Data File.

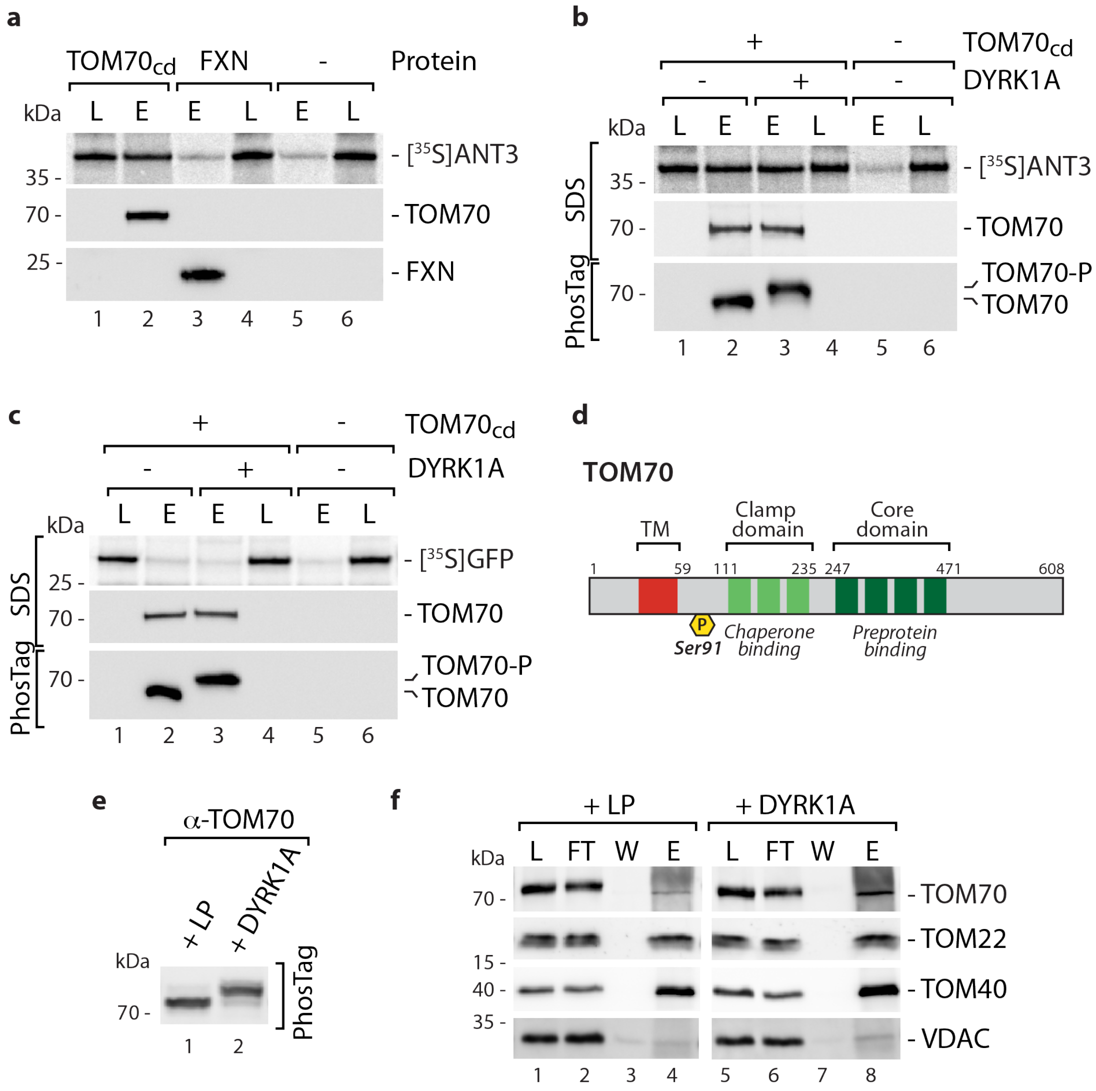

Supplementary Figure 5 | Binding assay for mitochondrial precursor proteins to TOM70 receptor domain and immunoprecipitation of the TOM complex. a, Binding assay of [^{35}S]ANT3 precursor in the presence of the soluble receptor domain of human TOM70 (TOM70$_{cd}$, lanes 1 and 2) and as a non-receptor protein control human Frataxin (FXN, lanes 3 and 4), both immobilized to Ni-NTA matrix via deca-His tag. **-** , binding assay in the absence of a bound protein (= background binding activity; lanes 5 and 6). **b**, Binding assay of [^{35}S]ANT3 precursor in the presence or absence of TOM70$_{cd}$. Where indicated TOM70$_{cd}$ was incubated with DYRK1A. Phos-tag electrophoresis (lower panel) shows DYRK1A specific phosphorylation of TOM70$_{cd}$ as control. **c**, Binding assay as in (**b**) but with [^{35}S]GFP as precursor protein. L, load/input of precursor protein (20% of elution); E, elution. **d**, Domain structure of human TOM70 indicating position of phosphorylated residue Ser91. Boxes indicate TPR (*tetratricopeptide repeat motif*) segments (modified from Refs 17 and 30). **e**, Phos-tag gels of mitochondria that were incubated before in the presence of lambda phosphatase (LP, TOM70 dephosphorylated) or DYRK1A (TOM70 phosphorylated). **f**, Immunoprecipitation of TOM complex via TOM22 antibodies from HEK293T mitochondria that were treated as in **e**. Samples were analyzed by SDS-PAGE followed by immunoblotting with indicated antibodies. L, load; FT, flowthrough; W, wash; E, elution. Unprocessed immunoblots and autoradiography scans are reported in the Source Data File.

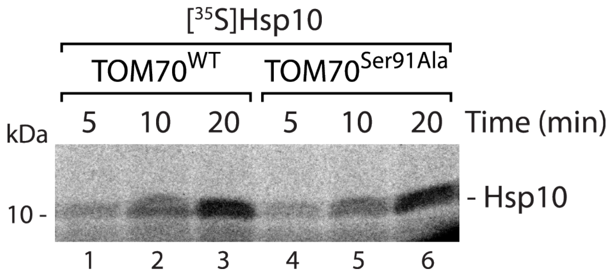

Supplementary Figure 6 | Presequence import pathway is not affected by TOM70^{Ser91Ala}. Import of radiolabelled Hsp10 into mitochondria isolated from TOM70$_{KD}$ cells re-expressing TOM70^{WT} or TOM70^{Ser91Ala}. Samples were treated with Proteinase K and analyzed after SDS-PAGE by autoradiography. The unprocessed autoradiography scan is reported in the Source Data File.

Supplementary Table 1

Primary antibodies used in this study. Antisera were diluted in 1×TBS with 5% (w/v) milk powder.

Antigen	Dilution	Reference/Company
14-3-3	1:2500	Santa Cruz Biotechnology sc-1657
DYRK1A	1:500	Sigma-Aldrich D1819
DYRK1B	1:500	Cell signaling 2703S
His	1:1000	Qiagen 34660
FXN	1:250	GR5190-4
TOM20	1:250	GR5002-4
TOM22	1:250	GR2152-3/4
TOM40	1:1500	Proteintech 18409-1-AP
TOM70	1:250	GR5280-4 or GR5005-3
TOM70 pS91	1:50	Eurogentec DE19042
VDAC(3)	1:250	GR1514-7

Supplementary Table 2
List of primers for RT-qPCR used in this study.

**Name**	**5' to 3' sequence**
ACTB fw	GCACTCTTCCAGCCTTCCTT
ACTB rv	AATGCCAGGGTACATGGTGG
CLK2 fw	GAAGTCGCTCCTGGTCAAGT
CLK2 rv	TGCTTCGAGAACGGACATGG
DYRK1A fw	TTCATTTGGACAGGTTGTGAAAG
DYRK1A rv	CGCACTTCTATCTGGGCTTGAT
DYRK1B fw	GCCAGGTGGTGAAAGCCTAT
DYRK1B rv	ACAGGTTGTAGGACAGCAGC
DYRK2 fw	GTCTGCCTTTGGTTCGCAAG
DYRK2 rv	ACACGCTGATGCTCGTAACA
DYRK3 fw	CAGCAGCGGAGGTTGGG
DYRK3 rv	TCTTCTGGGTGGAGGTGGTT
HIPK1 fw	AGCCAGAACCAGCAGTCATC
HIPK1 rv	TGCCATGCTGGAAGGTGTAG
HIPK2 fw	GCCAGAGCAAGAACATACCA
HIPK2 rv	CGACGCATTAGGTTGTGTGG
TOMM20 fw	CCGCAAAAGACGAAGTGACC
TOMM20 rv	GGTAACTTGGAAAGCCCAGC
TOMM40 fw	CCAACTACCACTTCGGGGTC
TOMM40 rv	AGCTGGTGAATGACCTGAGC
TOMM70 fw	GCCTTTGCTGTCCACTCAAG
TOMM70 rv	AGCTGTCCTCGGTGGTGATA
