# Supplementary material for: Global kinome profiling reveals DYRK1A as critical activator of the human mitochondrial import machinery
Source: Nat Commun. 2021 Jul 13;12:4284. doi: 10.1038/s41467-021-24426-9 (PMC8277783; doi:10.1038/s41467-021-24426-9)
Supplement: Supplementary file 3 — Reporting Summary [file 41467_2021_24426_MOESM3_ESM.pdf]

nature research

Corresponding author(s): Chris Meisinger

Last updated by author(s): 2021/May/25

Reporting Summary

Nature Research wishes to improve the reproducibility of the work that we publish. This form provides structure for consistency and transparency in reporting. For further information on Nature Research policies, see our Editorial Policies and the Editorial Policy Checklist.

Statistics

For all statistical analyses, confirm that the following items are present in the figure legend, table legend, main text, or Methods section.

n/a	Confirmed	
☐	☒	The exact sample size (*n*) for each experimental group/condition, given as a discrete number and unit of measurement
☒	☐	A statement on whether measurements were taken from distinct samples or whether the same sample was measured repeatedly
☐	☒	The statistical test(s) used AND whether they are one- or two-sided<br>*Only common tests should be described solely by name; describe more complex techniques in the Methods section.*
☒	☐	A description of all covariates tested
☒	☐	A description of any assumptions or corrections, such as tests of normality and adjustment for multiple comparisons
☐	☒	A full description of the statistical parameters including central tendency (e.g. means) or other basic estimates (e.g. regression coefficient) AND variation (e.g. standard deviation) or associated estimates of uncertainty (e.g. confidence intervals)
☐	☒	For null hypothesis testing, the test statistic (e.g. *F*, *t*, *r*) with confidence intervals, effect sizes, degrees of freedom and *P* value noted<br>*Give P values as exact values whenever suitable.*
☒	☐	For Bayesian analysis, information on the choice of priors and Markov chain Monte Carlo settings
☒	☐	For hierarchical and complex designs, identification of the appropriate level for tests and full reporting of outcomes
☒	☐	Estimates of effect sizes (e.g. Cohen's *d*, Pearson's *r*), indicating how they were calculated

Our web collection on statistics for biologists contains articles on many of the points above.

Software and code

Policy information about availability of computer code

Data collection	No software was used.
Data analysis	ImageJ 1.52 software (Wayne Rasband, National Institutes of Health, USA); MaxQuant (version 1.6.2.10; https://maxquant.net/maxquant/); Perseus (v1.6.15.0; https://maxquant.net/perseus/); Multi Gauge (Fuji, V3.2); Image Analyzer LAS-4000 (Fuji Image Reader V1.12); Proteome Discoverer (v1.4); Mascot (v2.4)

For manuscripts utilizing custom algorithms or software that are central to the research but not yet described in published literature, software must be made available to editors and reviewers. We strongly encourage code deposition in a community repository (e.g. GitHub). See the Nature Research guidelines for submitting code & software for further information.

Data

Policy information about availability of data

All manuscripts must include a data availability statement. This statement should provide the following information, where applicable:

- Accession codes, unique identifiers, or web links for publicly available datasets
- A list of figures that have associated raw data
- A description of any restrictions on data availability

MS/MS data are deposited to the ProteomeXchange Consortium via the PRIDE partner repository (https://www.ebi.ac.uk/pride) with the dataset identifier PXD019520. Reviewer account details: Username: reviewer44210@ebi.ac.uk, Password: WEsMC5UM. All other data are available in the main article or the Supplementary information files. Source data and uncropped versions of all blots and gels are provided in the Source Data file. Further data and resources from this study are available from the corresponding author upon reasonable request.

Field-specific reporting

Please select the one below that is the best fit for your research. If you are not sure, read the appropriate sections before making your selection.

☒ Life sciences ☐ Behavioural & social sciences ☐ Ecological, evolutionary & environmental sciences

For a reference copy of the document with all sections, see nature.com/documents/nr-reporting-summary-flat.pdf

Life sciences study design

All studies must disclose on these points even when the disclosure is negative.

Sample size	Sample sizes were not chosen based on pre-specified effect size, but were selected based on the commonly applied standards in the field. This resulted in statistically meaningful comparisons. Multiple independent experiments were carried out and all biochemical experiments were performed in biological replicates with n= or >3. Our previous studies have shown robust consistency between our assays, so that n=3 or n>3 are established as sufficient to reveal differences between our samples. Information about sample size is provided in the figure legends and in the Statistics and Reproducibility paragraph of the methods section.
Data exclusions	No samples were excluded from the analysis.
Replication	All experiments were carried out under clearly defined and standard conditions. All attempts of replication were successful. The number of replicates of each experiment is specified in the corresponding figure legend and detailed in the statistics and reproducibility section within the Methods chapter.
Randomization	Not relevant to this study. All relevant experiments were performed by at least two or more researchers and included positive and negative controls. Regarding animals used for this study: Only tissues from wild-type mice were used, therefore, randomization was not possible. No randomization for other experiments using cultured cell lines was necessary as all cells were from the same parental cell dish and passaged for the same number.
Blinding	Blinding was applied for life cell imaging analysis and included blinding during data collection. Cells for biochemical assays (in organello import, immunoblots) were not collected or processed blindly as knowledge of the treatment of each sample was necessary for data generation. Similarly, data analysis was performed from the person who conducted the experiment in these cases and therefore he/she was aware of the conditions that were analysed.

Reporting for specific materials, systems and methods

We require information from authors about some types of materials, experimental systems and methods used in many studies. Here, indicate whether each material, system or method listed is relevant to your study. If you are not sure if a list item applies to your research, read the appropriate section before selecting a response.

Materials & experimental systems

n/a	Involved in the study	
☐	☒	Antibodies
☐	☒	Eukaryotic cell lines
☒	☐	Palaeontology and archaeology
☐	☒	Animals and other organisms
☒	☐	Human research participants
☒	☐	Clinical data
☒	☐	Dual use research of concern

Methods

n/a	Involved in the study	
☒	☐	ChIP-seq
☒	☐	Flow cytometry
☒	☐	MRI-based neuroimaging

Antibodies

Antibodies used

All antibodies used in this study are listed with detailed information in Supplementary Table 1.
Primary antibodies:
14-3-3: dilution 1:2500; Santa Cruz Biotechnology sc-1657; LOT#L3015
DYRK1A: dilution 1:500; Sigma-Aldrich, Cat#D1819; LOT#048K4807
DYRK1B: dilution 1:500; Cell signaling 2703S; LOT#2
His: dilution 1:1000; Qiagen 34660
FXN: dilution 1:250; GR5190-4
TOM20: dilution 1:250; GR5002-4
TOM22: dilution 1:250; GR2152-3/4
TOM40: dilution 1:1500; Proteintech 18409-1-AP; LOT#00040008
TOM70: dilution 1:250; GR5280-4 or GR5005-3
TOM70 pS91: dilution 1:50; Eurogentec DE19042
VDAC(3): dilution 1:250; GR1514-7

Validation	Antibodies were purchased from suppliers with the following authentification data:<br>14-3-3 antibody: https://www.scbt.com/p/pan-14-3-3-antibody-h-8<br>DYRK1A antibody: https://www.sigmaaldrich.com/catalog/product/sigma/d1819?lang=de&region=DE#productDetailSafetyRelatedDocs<br>DYRK1B antibody: https://www.cellsignal.de/products/primary-antibodies/dyrk1b-antibody/2703?_=1621932902047&Ntt=2703&tahead=true<br>TOM40 antibody: https://www.ptglab.com/products/TOMM40-Antibody-18409-1-AP.htm<br>The validation of custom-manufactured rabbit polyclonal anti-VDAC can be found in following publication:<br>Vögtle et al. (2018). Mutations in PMPCB Encoding the Catalytic Subunit of the Mitochondrial Presequence Protease Cause Neurodegeneration in Early Childhood. Am. J. Hum. Genet. 102, 557-573.<br>Validation of custom-manufactured rabbit polyclonal anti-FXN was performed by detection of the purified protein after expression in E.coli and by immunoblots using isolated mitochondria from wild-type and FXN deletion cells.<br>For human TOM complex antibodies sera were validated by detection of the purified protein/cytosolic domain expressed in E.coli. Furthermore, immunoprecipitation with antisera directed against the receptor TOM22 under native conditions was performed followed by analysis of the eluate on SDS- and BN-PAGE. The native TOM complex of approximately 400 kDa was detected on the native gels and the antibodies against other TOM subunits were detected specifically in the elution on the SDS-PAGE when TOM22 antisera was used, but were absent in the control (pre-immune serum). TOM70 pS91 antibody was validated using WT and S91A variants of TOM70cd both treated with DYRK1A and ATP. Only WT but not S91A variant was specifically phosphorylated (additionally validated via MW size shift on PhosTag gel).

Eukaryotic cell lines

Policy information about cell lines

Cell line source(s)	Human embryonic kidney cell lines HEK293T were purchased from ATCC. Human U2OS cells were a kind gift from the laboratory of Dr. D. Slade (Max Perutz Labs, Vienna) and were purchased by Invitrogen.
Authentication	Cell lines from ATCC and Invitrogen are regularly authenticated and were used by us without further authentication.
Mycoplasma contamination	Cell lines were routinely tested for Mycoplasma contamination every second month and were not contaminated.
Commonly misidentified lines<br>(See ICLAC register)	No commonly misidentified cell lines were used in this study.

Animals and other organisms

Policy information about studies involving animals; ARRIVE guidelines recommended for reporting animal research

Laboratory animals	Male C57Bl6/N mice (8 weeks of age).
Wild animals	No wild animals were used in this study.
Field-collected samples	No field collected samples were used in this study.
Ethics oversight	Tissue sampling from sacrificed mice was approved by the government commission for animal protection and the ethics committee (University Medical Center of Freiburg University; X-18/10C).

Note that full information on the approval of the study protocol must also be provided in the manuscript.
