# Supplementary material for: Global kinome profiling reveals DYRK1A as critical activator of the human mitochondrial import machinery
Source: Nat Commun. 2021 Jul 13;12:4284. doi: 10.1038/s41467-021-24426-9 (PMC8277783; doi:10.1038/s41467-021-24426-9)

Figure 1b

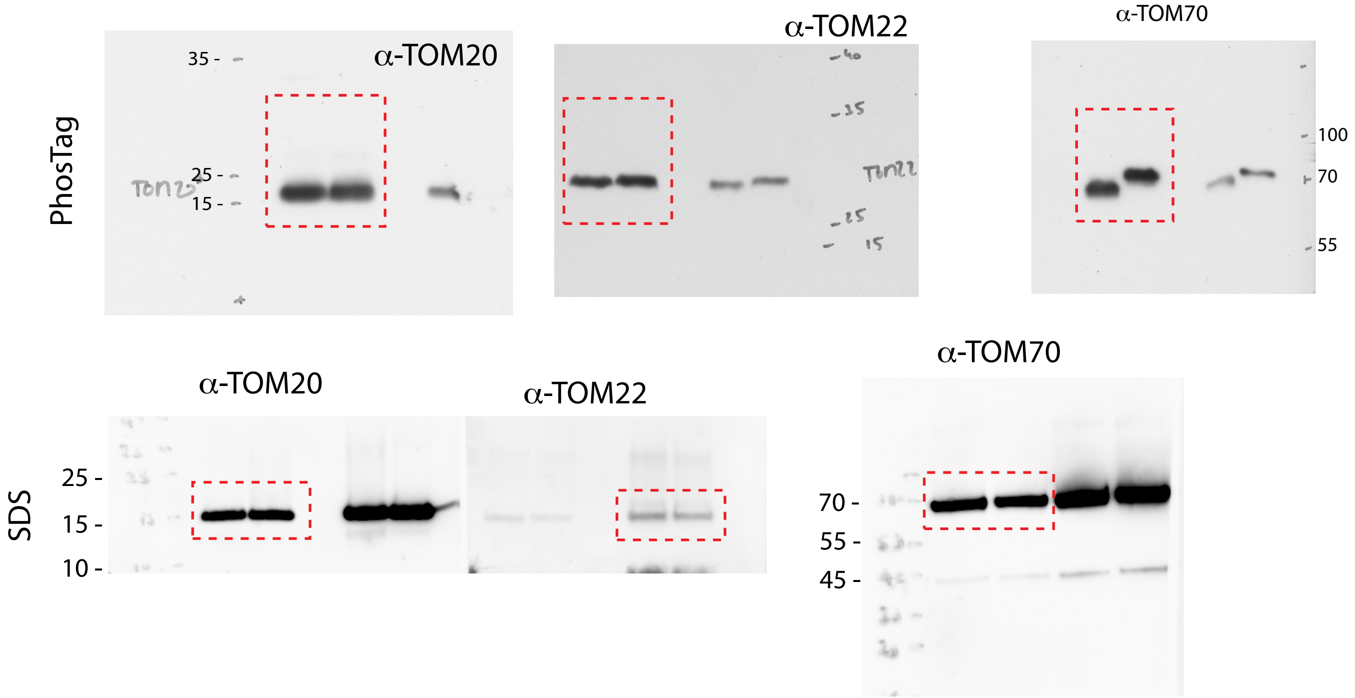

Figure 1c

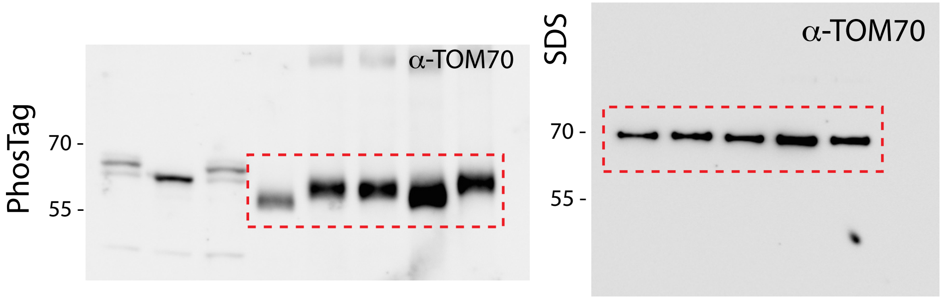

Figure 1d

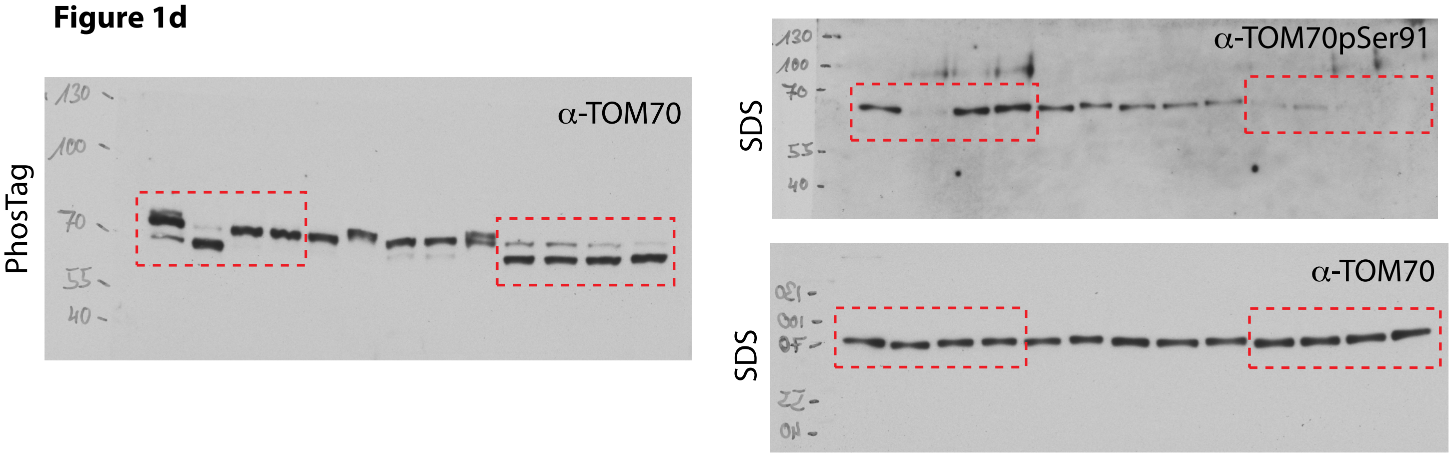

Figure 1e

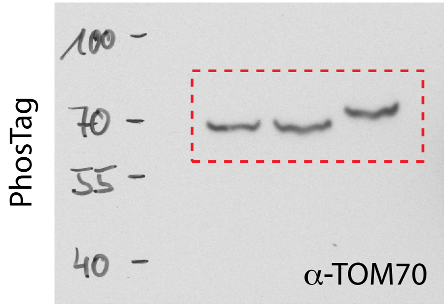

Figure 3a

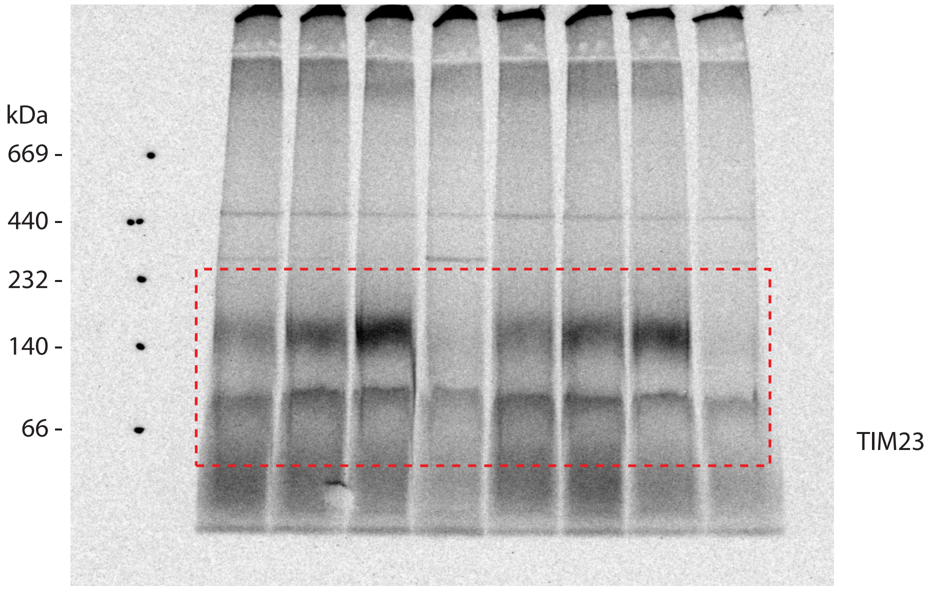

Figure 3b

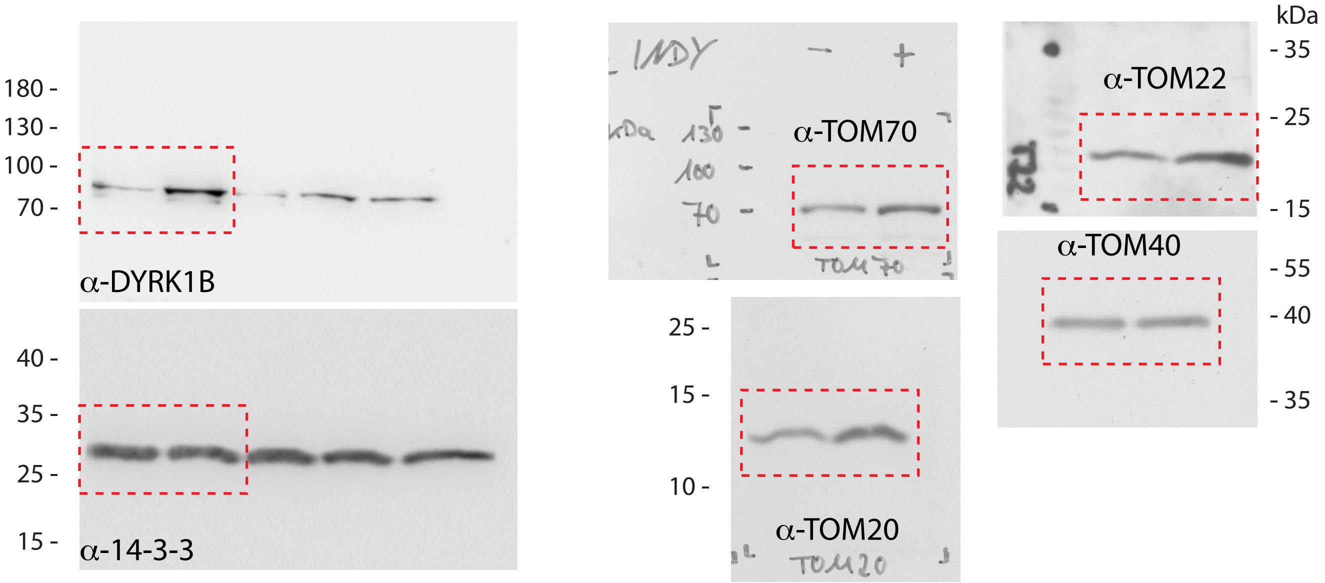

Figure 3d

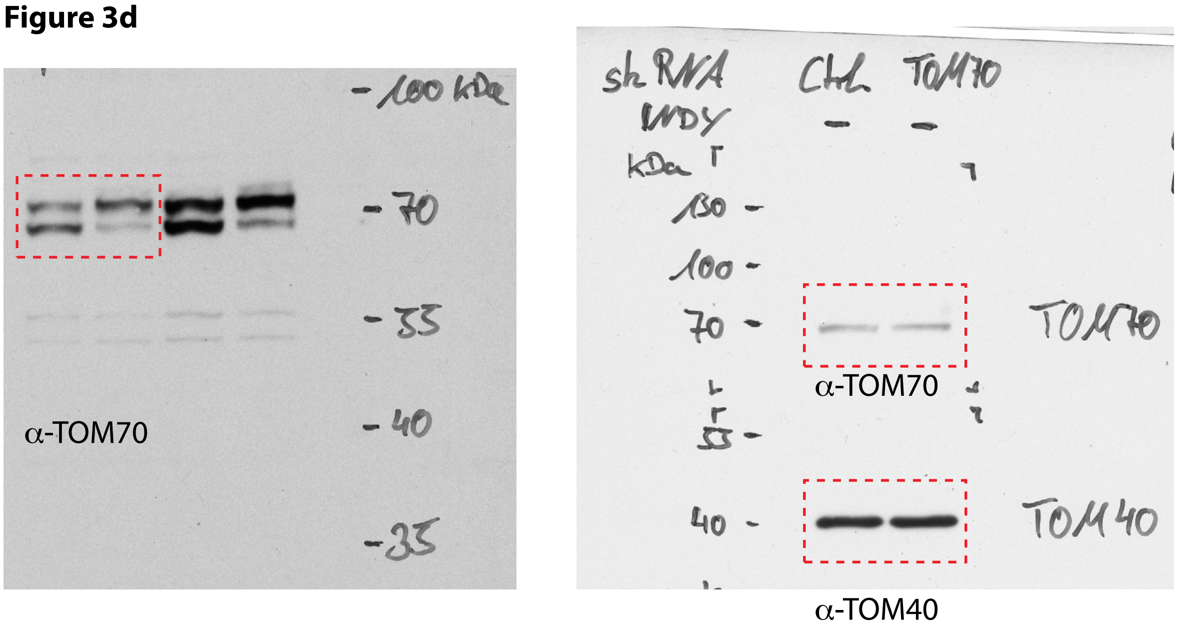

Figure 3e

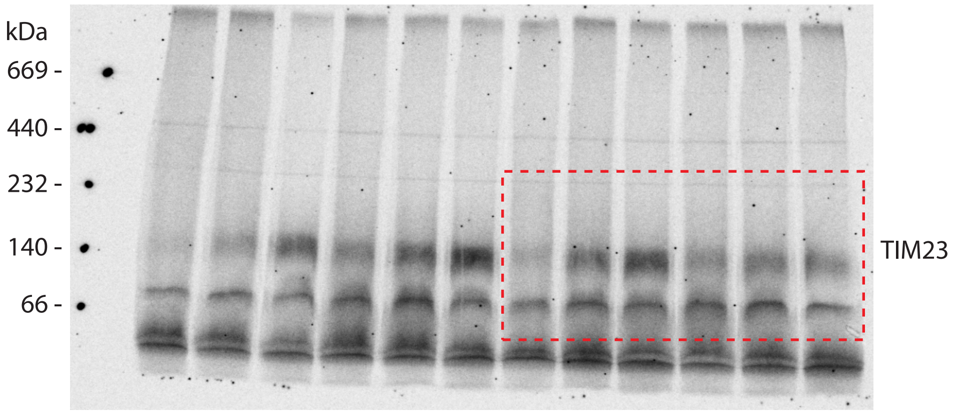

Figure 4a

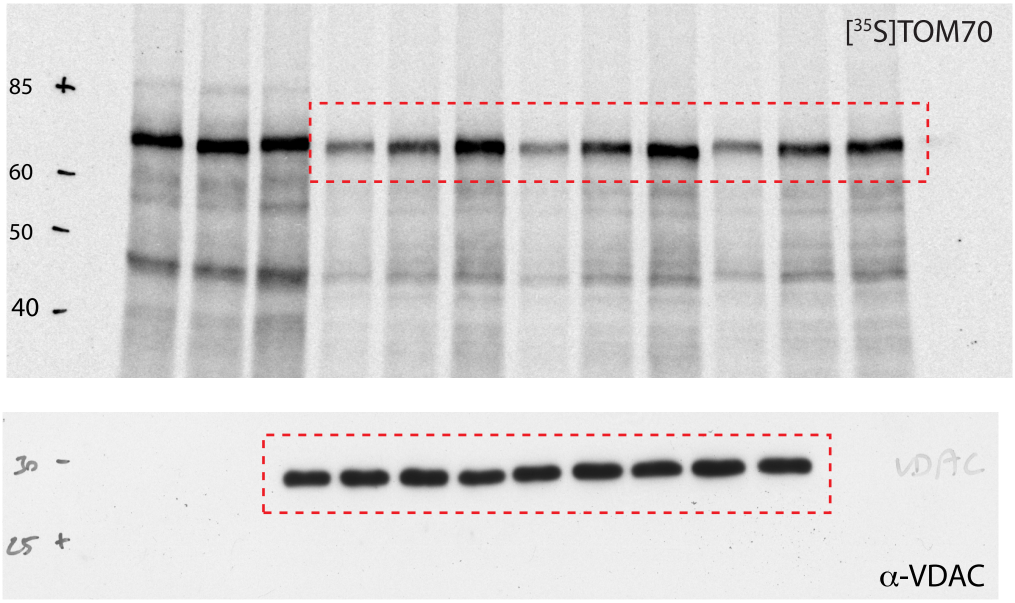

Figure 4b

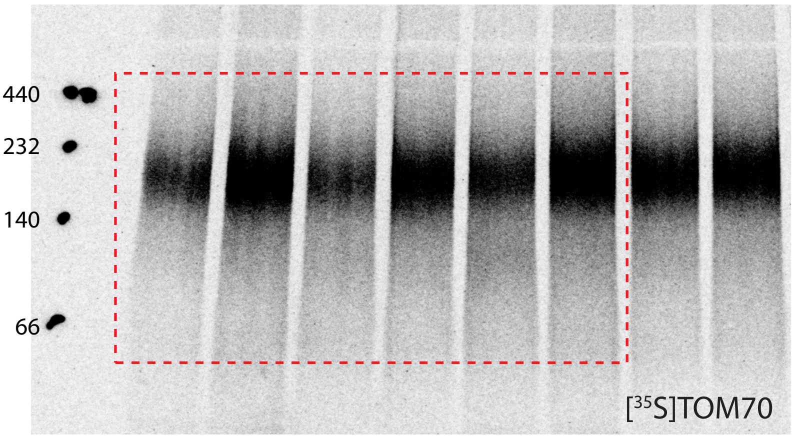

Figure 4d

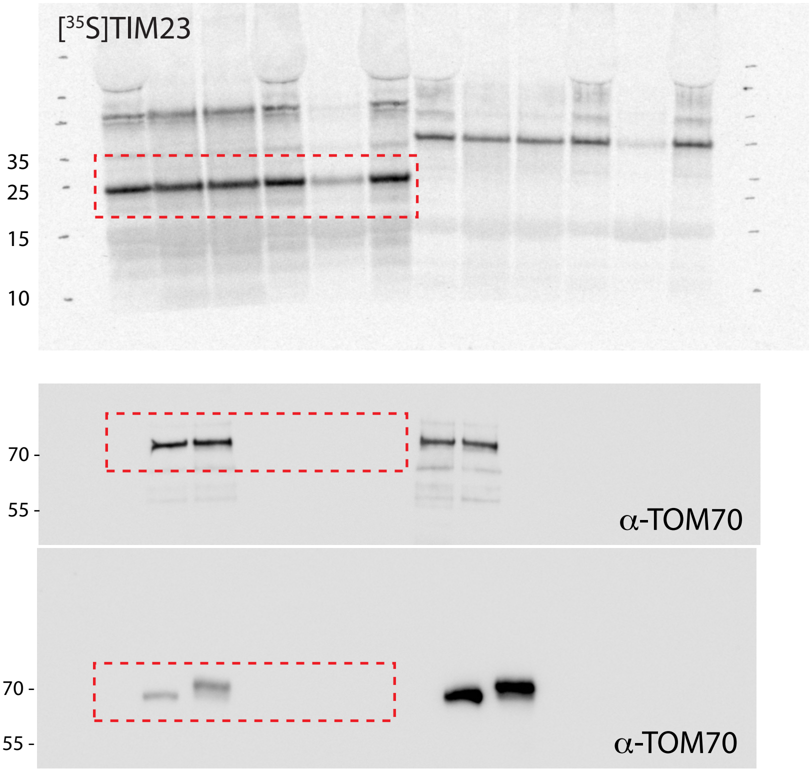

Figure 4e

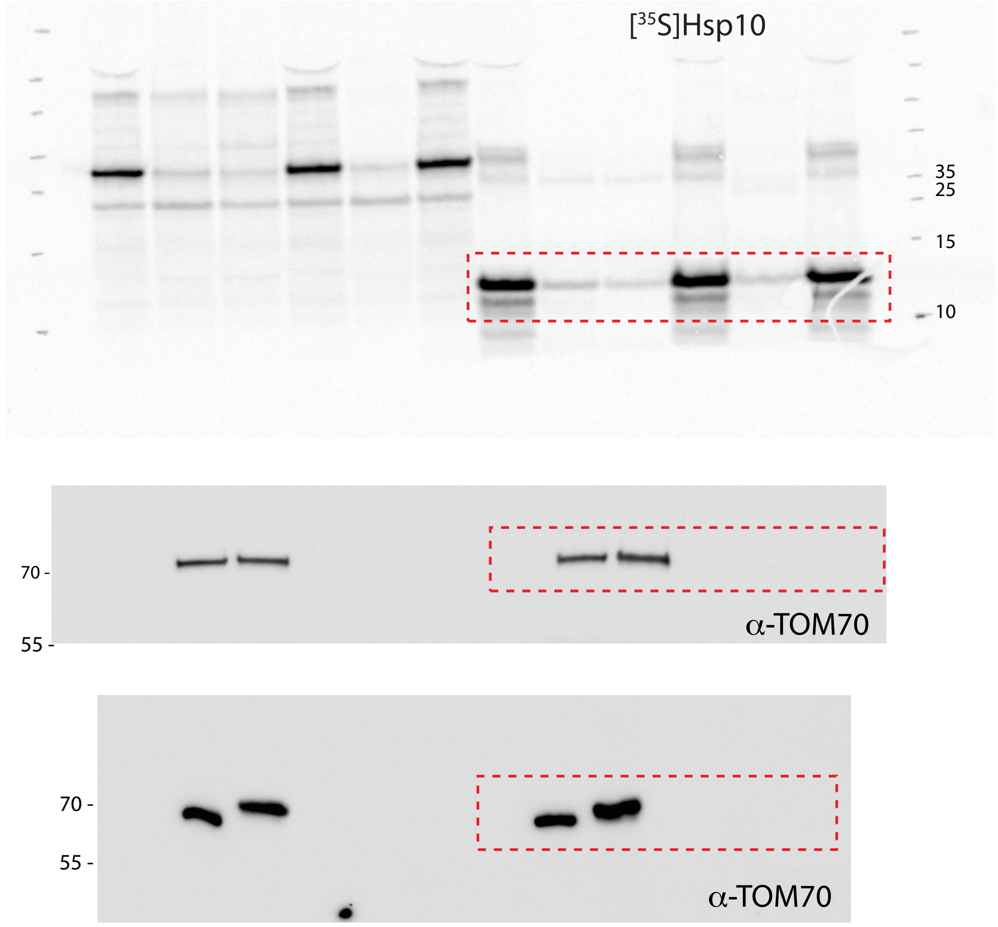

Figure 4g

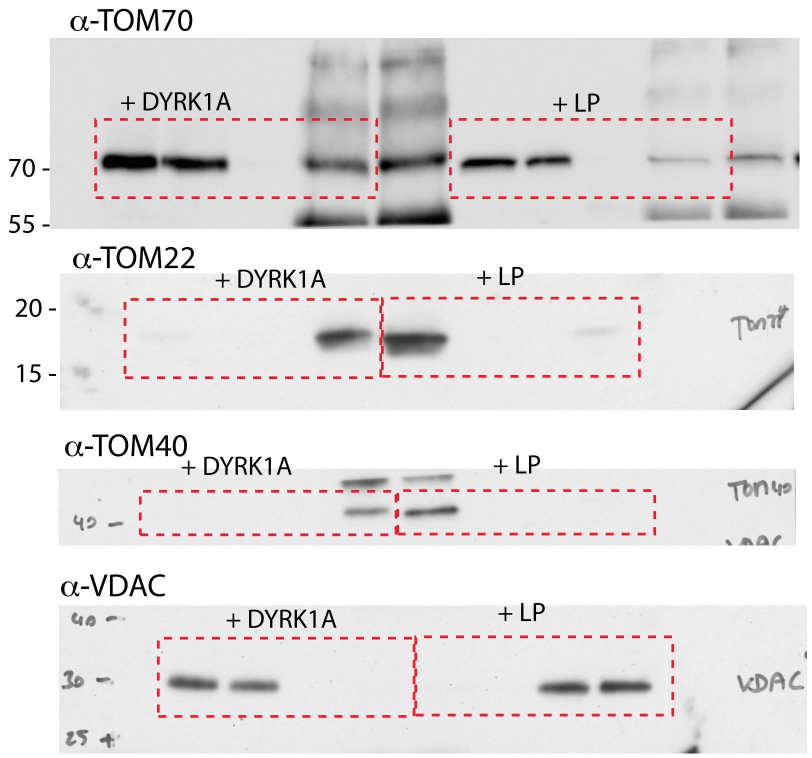

Figure 4h

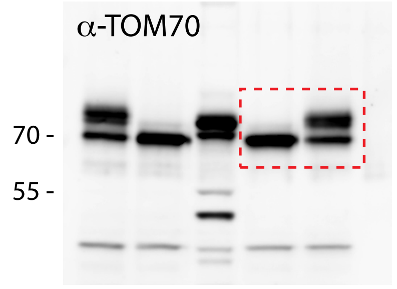

Figure 5a

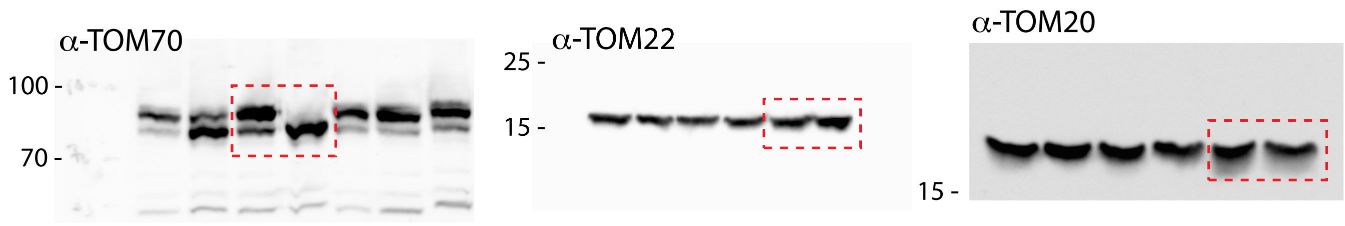

Figure 5b

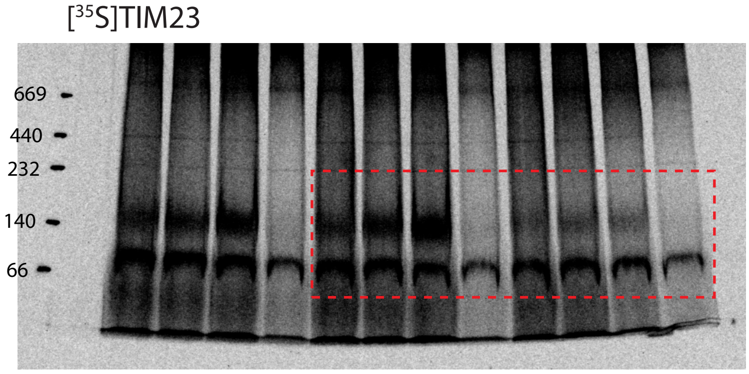

Figure 5d

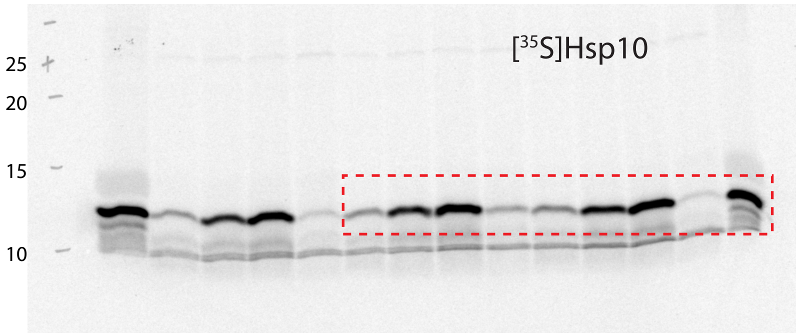

Figure 5e

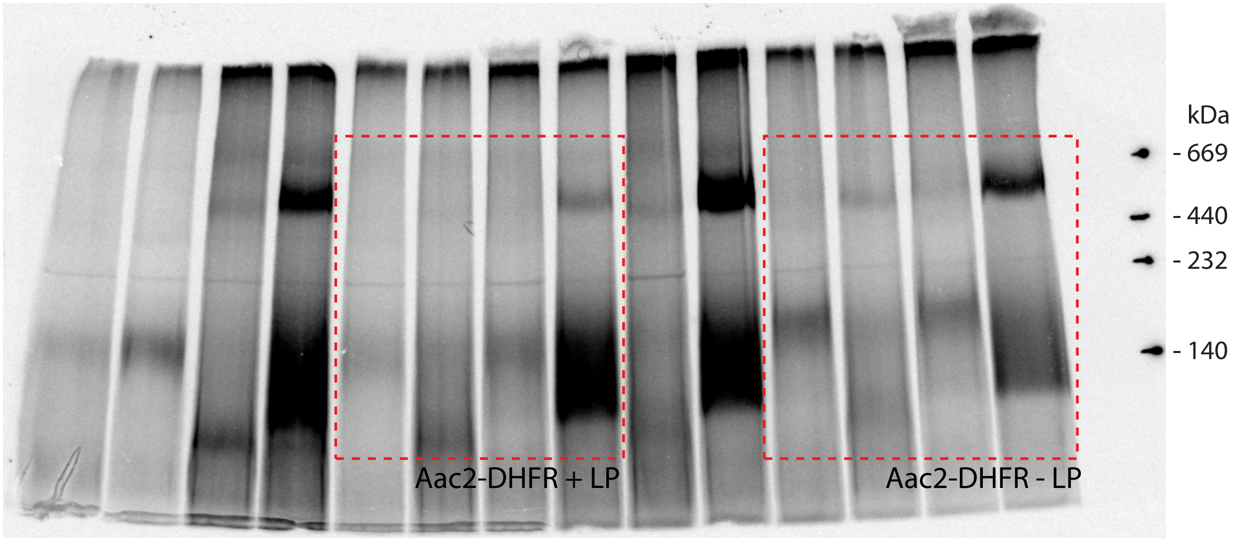

Figure 5f

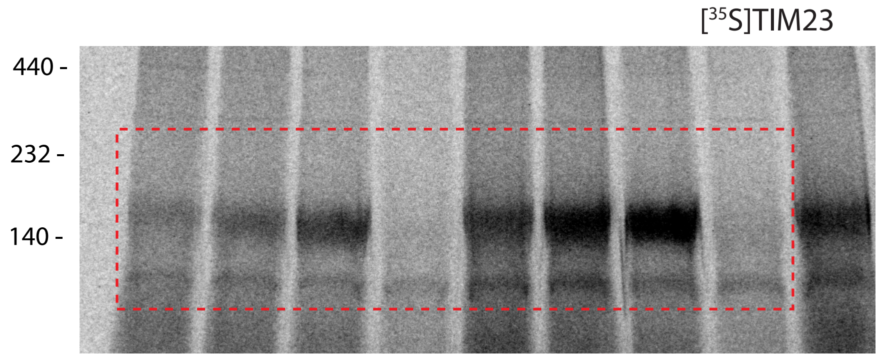

Figure 5g

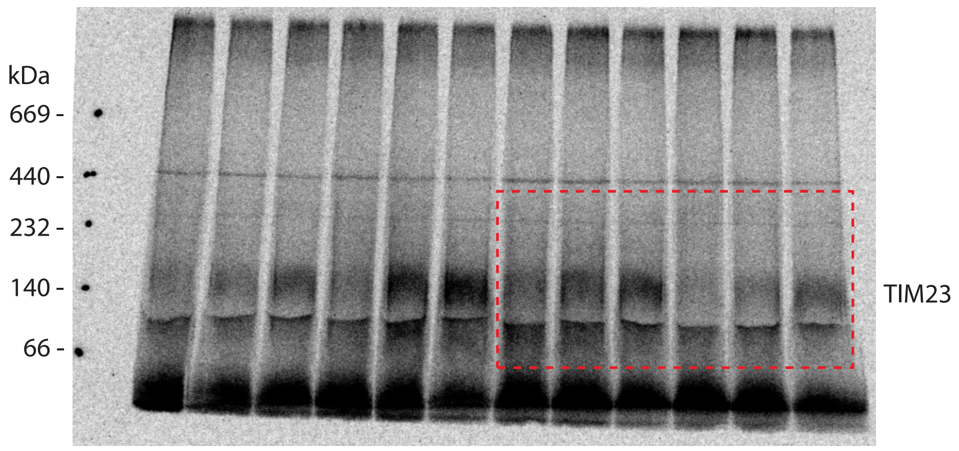

Figure 1a Supplementary Figure

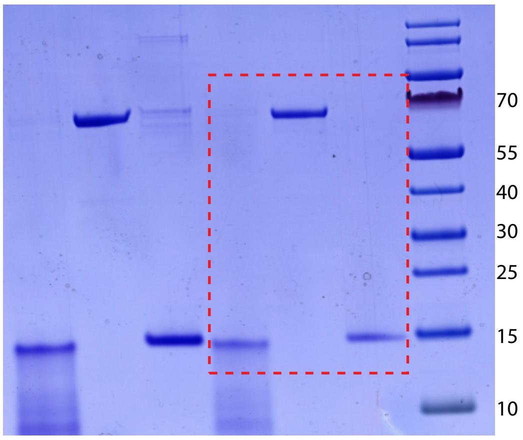

Figure 1d Supplementary Figure

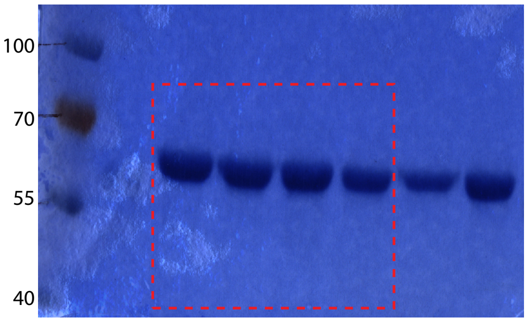

Figure 1e Supplementary Figure

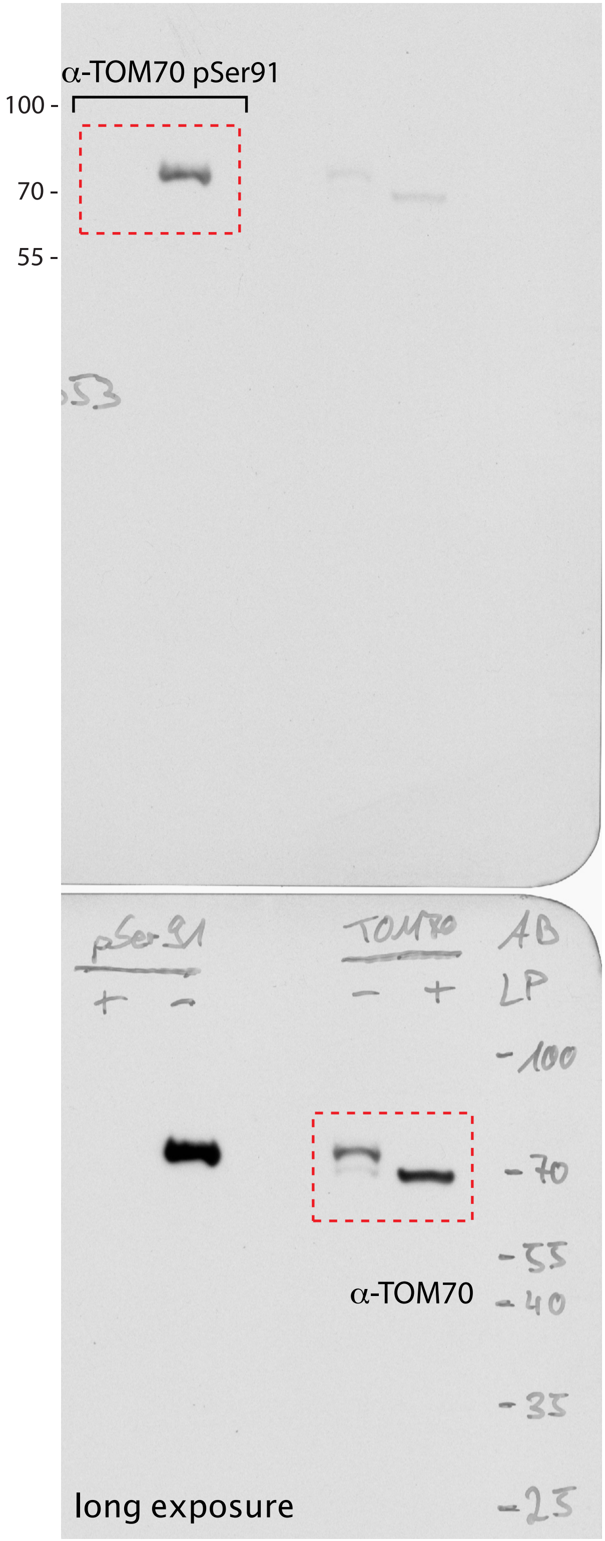

Figure 1f Supplementary Figure

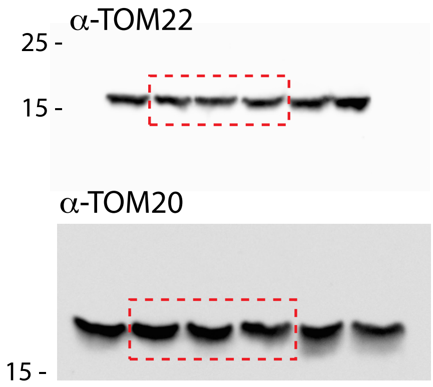

Figure 1h Supplementary Figure

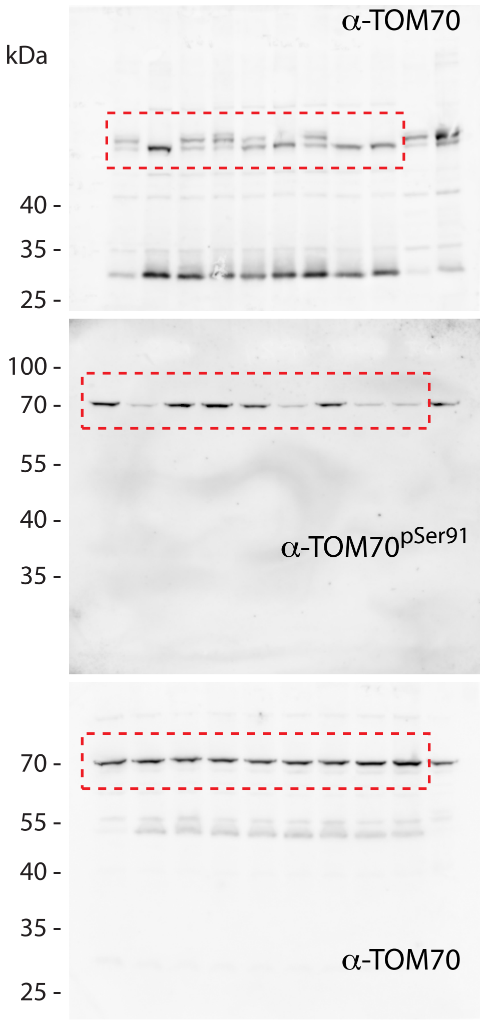

Figure 2a Supplementary Figure

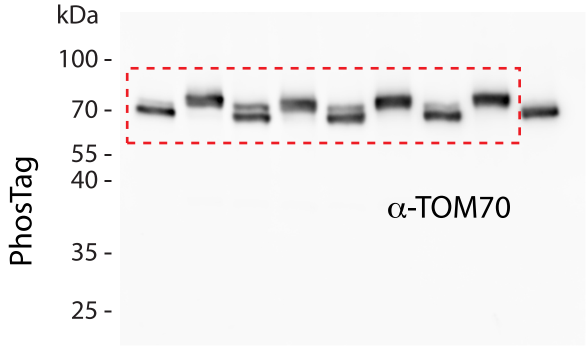

Figure 2b Supplementary Figure

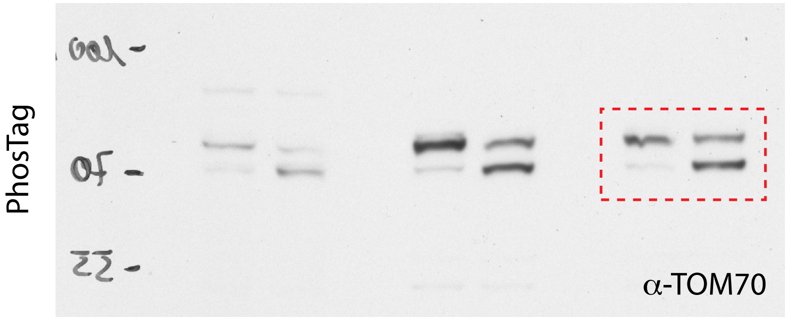

Figure 4b Supplementary Figure

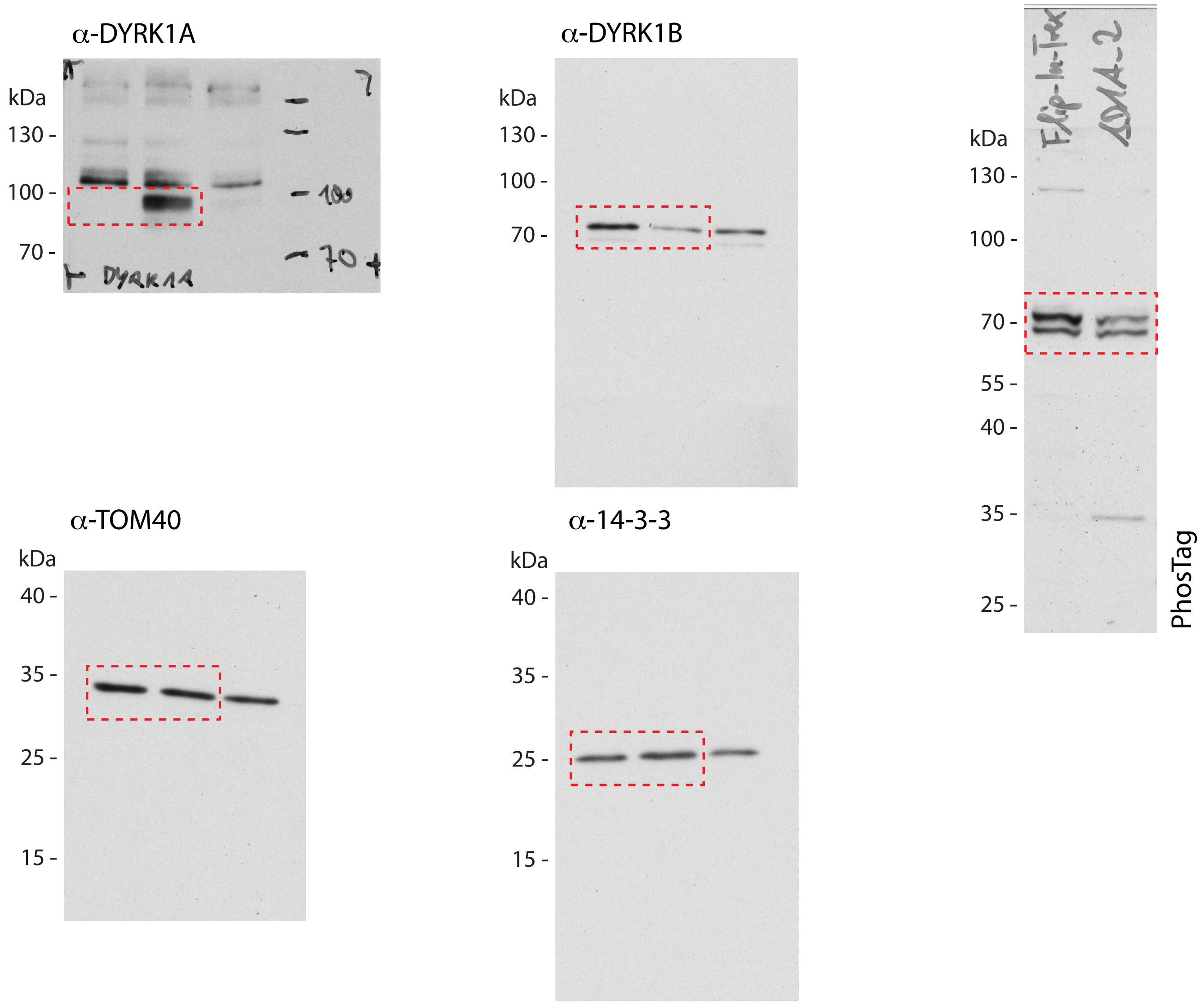

Figure 4c Supplementary Figure

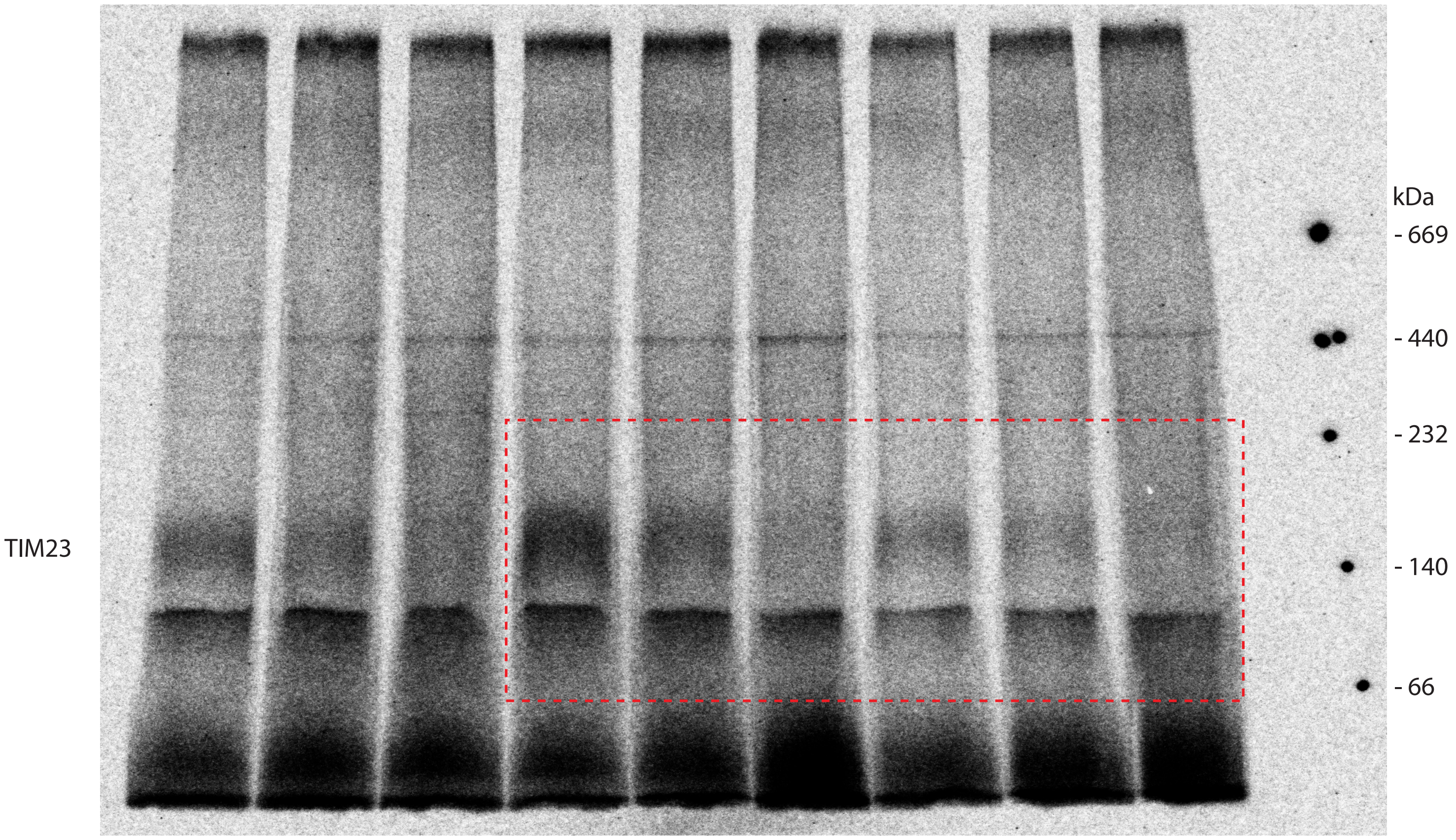

Figure 5a Supplementary Figure

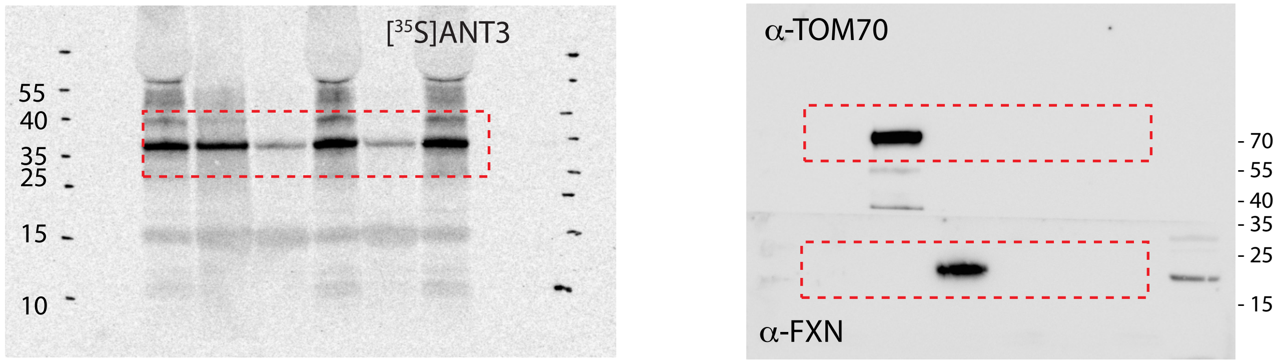

Figure 5b Supplementary Figure

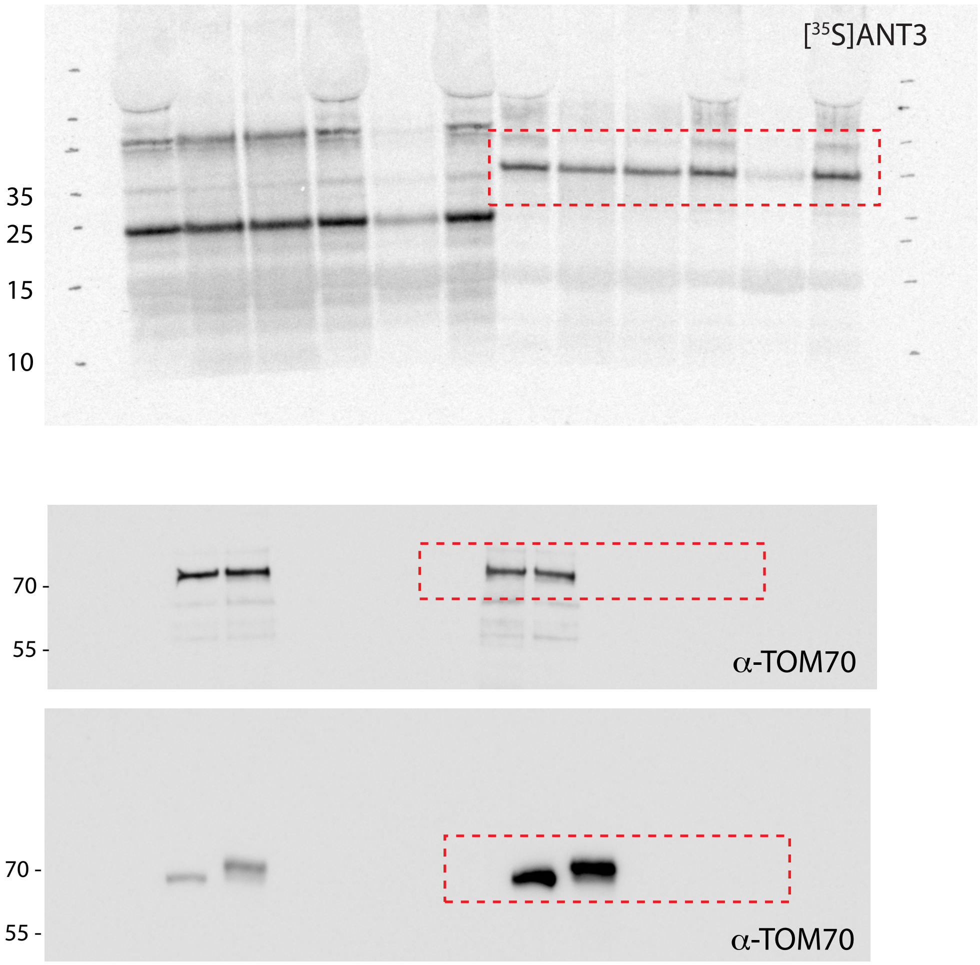

Figure 5c Supplementary Figure

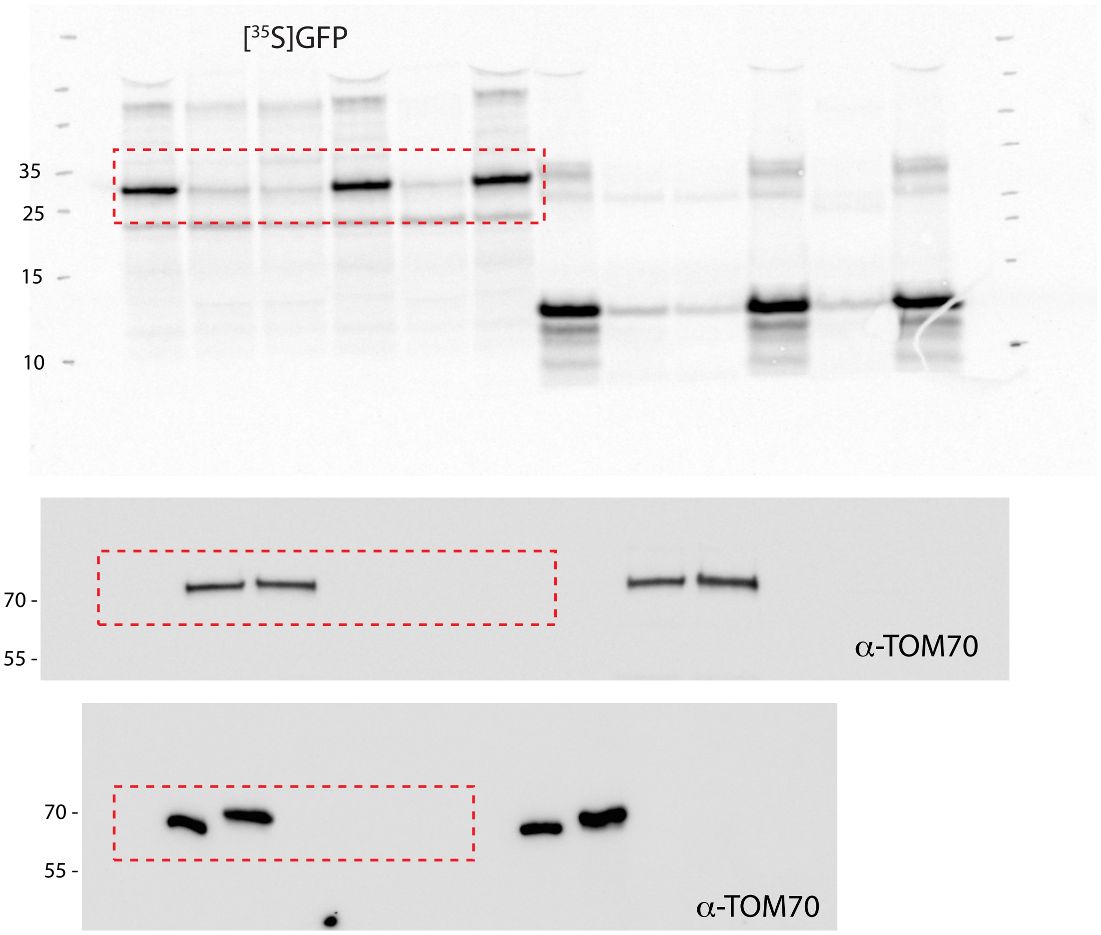

Figure 5e Supplementary Figure

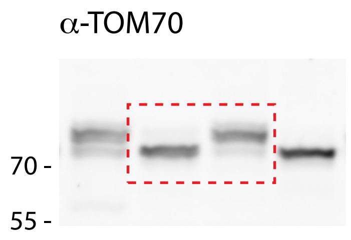

Figure 5f Supplementary Figure

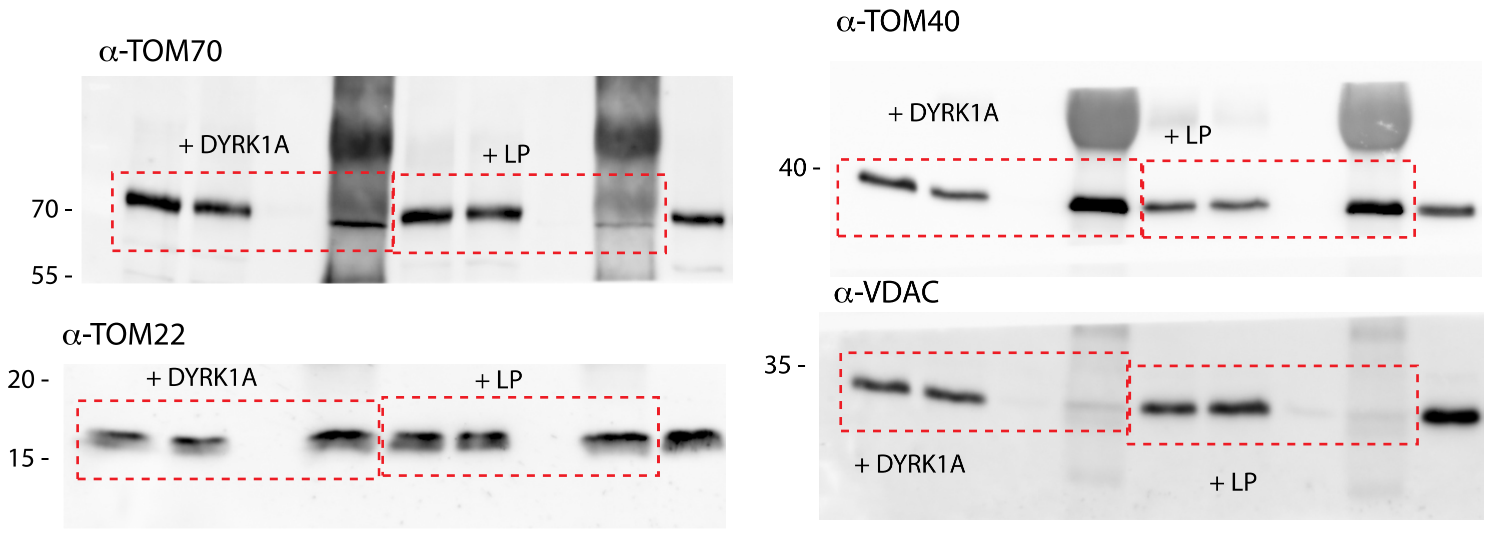

Figure 6 Supplementary Figure

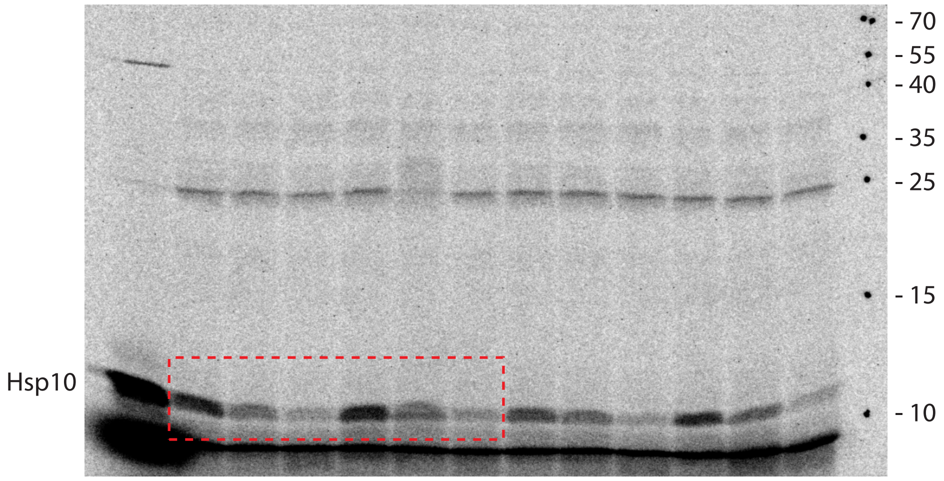

Supplement: Supplementary file 4 — Source Data [file 41467_2021_24426_MOESM4_ESM.zip › SourceDataFiles/Source Data file_Walter and Marada et al.pdf]
